# Childhood Sexual Abuse and the Development of Recurrent Major Depression in Chinese Women

**DOI:** 10.1371/journal.pone.0087569

**Published:** 2014-01-29

**Authors:** Jing Chen, Yiyun Cai, Enzhao Cong, Ying Liu, Jingfang Gao, Youhui Li, Ming Tao, Kerang Zhang, Xumei Wang, Chengge Gao, Lijun Yang, Kan Li, Jianguo Shi, Gang Wang, Lanfen Liu, Jinbei Zhang, Bo Du, Guoqing Jiang, Jianhua Shen, Zhen Zhang, Wei Liang, Jing Sun, Jian Hu, Tiebang Liu, Xueyi Wang, Guodong Miao, Huaqing Meng, Yi Li, Chunmei Hu, Yi Li, Guoping Huang, Gongying Li, Baowei Ha, Hong Deng, Qiyi Mei, Hui Zhong, Shugui Gao, Hong Sang, Yutang Zhang, Xiang Fang, Fengyu Yu, Donglin Yang, Tieqiao Liu, Yunchun Chen, Xiaohong Hong, Wenyuan Wu, Guibing Chen, Min Cai, Yan Song, Jiyang Pan, Jicheng Dong, Runde Pan, Wei Zhang, Zhenming Shen, Zhengrong Liu, Danhua Gu, Xiaoping Wang, Xiaojuan Liu, Qiwen Zhang, Yihan Li, Yiping Chen, Kenneth S. Kendler, Shenxun Shi, Jonathan Flint

**Affiliations:** 1 Shanghai Mental Health Center, Shanghai Jiaotong University School of Medicine, Shanghai, People's Republic of China; 2 Huashan Hospital of Fudan University, Shanghai, People's Republic of China; 3 The First Hospital of China Medical University, Shenyang, Liaoning, People's Republic of China; 4 Chinese Traditional Hospital of Zhejiang, Hangzhou, Zhejiang, People's Republic of China; 5 No.1 Hospital of Zhengzhou University, Zhengzhou, Henan, People's Republic of China; 6 Xinhua Hospital of Zhejiang Province, Hangzhou, Zhejiang, People's Republic of China; 7 No.1 Hospital of Shanxi Medical University, Taiyuan, Shanxi, People's Republic of China; 8 ShengJing Hospital of China Medical University, Shenyang, Liaoning, People's Republic of China; 9 No. 1 Hospital of Medical College of Xian Jiaotong University, Xian, Shaanxi, People's Republic of China; 10 Jilin Brain Hospital, Siping, Jilin, People's Republic of China; 11 Mental Hospital of Jiangxi Province, Nanchang, Jiangxi, People's Republic of China; 12 Xian Mental Health Center, Xian, Shaanxi, People's Republic of China; 13 Beijing Anding Hospital of Capital University of Medical Sciences, Beijing, People's Republic of China; 14 Shandong Mental Health Center, Jinan, Shandong, People's Republic of China; 15 No. 3 Hospital of Sun Yat-sen University, Guangzhou, Guangdong, People's Republic of China; 16 Hebei Mental Health Center, Baoding, Hebei, People's Republic of China; 17 Chongqing Mental Health Center, Chongqing, People's Republic of China; 18 Tianjin Anding Hospital, Tianjin, People's Republic of China; 19 No.4 Hospital of Jiangsu University, Zhenjiang, Jiangsu, People's Republic of China; 20 Psychiatric Hospital of Henan Province, Xinxiang, Henan, People's Republic of China; 21 Nanjing Brain Hospital, Nanjing, Jiangsu, People's Republic of China; 22 Harbin Medical University, Haerbin, Heilongjiang, People's Republic of China; 23 Shenzhen Kang Ning Hospital, Shenzhen, Guangdong, People's Republic of China; 24 First Hospital of Hebei Medical University, Shijiazhuang, Hebei, People's Republic of China; 25 Guangzhou Brain Hospital (Guangzhou Psychiatric Hospital), Guangzhou, Guangdong, People's Republic of China; 26 No.1 Hospital of Chongqing Medical University, Chongqing, People's Republic of China; 27 Dalian No.7 Hospital, Dalian, Liaoning, People's Republic of China; 28 No.3 Hospital of Heilongjiang Province, Beian, Heilongjiang, People's Republic of China; 29 Wuhan Mental Health Center, Wuhan, Hubei, People's Republic of China; 30 Sichuan Mental Health Center, Mianyang, Sichuan, People's Republic of China; 31 Mental Health Institute of Jining Medical College, Jining, Shandong, People's Republic of China; 32 Liaocheng No.4 Hospital, Liaocheng, Shandong, People's Republic of China; 33 Mental Health Center of West China Hospital of Sichuan University, Chengdu, Sichuan, People's Republic of China; 34 Suzhou Guangji Hospital, Suzhou, Jiangsu, People's Republic of China; 35 Anhui Mental Health Center, Hefei, Anhui, People's Republic of China; 36 Ningbo Kang Ning Hospital, Ningbo, Zhejiang, People's Republic of China; 37 Changchun Mental Hospital, Changchun, Jilin, People's Republic of China; 38 No.2 Hospital of Lanzhou University, Lanzhou, Gansu, People's Republic of China; 39 Fuzhou Psychiatric Hospital, Fuzhou, Fujian, People's Republic of China; 40 Harbin No.1 Special Hospital, Haerbin, Heilongjiang, People's Republic of China; 41 Jining Psychiatric Hospital, Jining, Shandong, People's Republic of China; 42 No.2 Xiangya Hospital of Zhongnan University, Changsha, Hunan, People's Republic of China; 43 Xijing Hospital of No.4 Military Medical University, Xian, Shaanxi, People's Republic of China; 44 Mental Health Center of Shantou University, Shantou, Guangdong, People's Republic of China; 45 Tongji University Hospital, Shanghai, People's Republic of China; 46 Huaian No.3 Hospital, Huaian, Jiangsu, People's Republic of China; 47 Huzhou No.3 Hospital, Huzhou, Zhejiang, People's Republic of China; 48 Mudanjiang Psychiatric Hospital of Heilongjiang Province, Mudanjiang, Heilongjiang, People's Republic of China; 49 No.1 Hospital of Jinan University, Guangzhou, Guangdong, People's Republic of China; 50 Qingdao Mental Health Center, Qingdao, Shandong, People's Republic of China; 51 Guangxi Longquanshan Hospital, Liuzhou, Guangxi, People's Republic of China; 52 Daqing No.3 Hospital of Heilongjiang Province, Daqing, Heilongjiang, People's Republic of China; 53 Tangshan No.5 Hospital, Tangshan, Hebei, People's Republic of China; 54 Anshan Psychiatric Rehabilitation Hospital, Anshan, Liaoning, People's Republic of China; 55 Weihai Mental Health Center, Weihai, Shandong, People's Republic of China; 56 Renmin Hospital of Wuhan University, Wuhan, Hubei, People's Republic of China; 57 Tianjin First Center Hospital, Tianjin, People's Republic of China; 58 Hainan Anning Hospital, Haikou, Hainan, People's Republic of China; 59 Wellcome Trust Centre for Human Genetics, Oxford, United Kingdom; 60 Clinical Trial Service Unit, Oxford, United Kingdom; 61 Virginia Institute for Psychiatric and Behavioral Genetics, Department of Psychiatry, Virginia Commonwealth University, Richmond, Virginia, United States of America; Chinese Traditional Hospital of Zhejiang, China

## Abstract

**Background:**

Our prior study in Han Chinese women has shown that women with a history of childhood sexual abuse (CSA) are at increased risk for developing major depression (MD). Would this relationship be found in our whole data set?

**Method:**

Three levels of CSA (non-genital, genital, and intercourse) were assessed by self-report in two groups of Han Chinese women: 6017 clinically ascertained with recurrent MD and 5983 matched controls. Diagnostic and other risk factor information was assessed at personal interview. Odds ratios (ORs) were calculated by logistic regression.

**Results:**

We confirmed earlier results by replicating prior analyses in 3,950 new recurrent MD cases. There were no significant differences between the two data sets. Any form of CSA was significantly associated with recurrent MD (OR 4.06, 95% confidence interval (CI) [3.19–5.24]). This association strengthened with increasing CSA severity: non-genital (OR 2.21, 95% CI 1.58–3.15), genital (OR 5.24, 95% CI 3.52–8.15) and intercourse (OR 10.65, 95% CI 5.56–23.71). Among the depressed women, those with CSA had an earlier age of onset, longer depressive episodes. Recurrent MD patients those with CSA had an increased risk for dysthymia (OR 1.60, 95%CI 1.11–2.27) and phobia (OR 1.41, 95%CI 1.09–1.80). Any form of CSA was significantly associated with suicidal ideation or attempt (OR 1.50, 95% CI 1.20–1.89) and feelings of worthlessness or guilt (OR 1.41, 95% CI 1.02–2.02). Intercourse (OR 3.47, 95%CI 1.66–8.22), use of force and threats (OR 1.95, 95%CI 1.05–3.82) and how strongly the victims were affected at the time (OR 1.39, 95%CI 1.20–1.64) were significantly associated with recurrent MD.

**Conclusions:**

In Chinese women CSA is strongly associated with recurrent MD and this association increases with greater severity of CSA. Depressed women with CSA have some specific clinical traits. Some features of CSA were associated with greater likelihood of developing recurrent MD.

## Introduction

Studies carried out in Western populations indicated that childhood sexual abuse (CSA) increases the risk of developing major depression (MD) [1], [2], [3], [4], [5], [6], [7], [8], [9], [10], [11], [12], [13], [14], [15]. Furthermore, these studies also show that depressed patients who reported CSA differ in some clinical features from MD patients without a history of CSA. For example, they had been shown to have more severe depressive symptoms, longer duration of episodes, earlier onset, and higher levels of comorbidity [16], [17], [18], [19].

Some studies carried out in China reported higher levels of depressive symptoms in participants who experienced CSA [20]. However, none of these studies examined the association between CSA exposure and the risk of developing MD. The China, Oxford and VCU Experimental Research on Genetic Epidemiology (CONVERGE) study has been examining risk factors for MD reported in Western populations, using a large cohort of Chinese women with recurrent MD (rMD). We previously reported an analysis of CSA in a preliminary sample from this study consisting of 1,970 cases and 2,597 controls. Our findings were broadly similar to those seen in Western samples and can be summarized as follows: 1) Any form of CSA was significantly associated with rMD; this association strengthened with increasing CSA severity from non-genital, to genital, to intercourse. 2) The association between any form of CSA and rMD remained significant after accounting for parental history of depression, childhood emotional neglect (CEN), childhood physical abuse (CPA) and a measure of perceived parenting (derived from the parental bonding instrument (REF). 3) Among the depressed women, those with CSA had an earlier age of onset, longer depressive episodes and an increased risk for generalized anxiety disorder and dysthymia [21].

We now report analyses of CSA and rMD with our final sample of 5,983 controls and 6,017 cases. Our study had the following major aims. First, could we replicate our findings from our preliminary sample? Second, we examined in more detail the relationship between rMD and the nature of the CSA. In addition to the nature of the abuse, we asked which other features of CSA (including the age and gender of the perpetrator, and the relationship of the perpetrator to the victim) increased the risk of rMD. Third, we examined the association between CSA and individual depressive symptoms to find out whether the nature of the illness differed between those who have and those who did not have a history of CSA.

## Methods

### Sample

The data for the present study were drawn from the China, Oxford and VCU Experimental Research on Genetic Epidemiology (CONVERGE) study of MD. These analyses were based on a total of 6017 cases recruited from 58 provincial mental health centres and psychiatric departments of general medical hospitals in 45 cities and 23 provinces, and 5983 controls who were recruited from patients undergoing minor surgical procedures at general hospitals (37%) or from local community centres (63%). Where controls were obtained from a hospital, this was always from the same hospital as the cases.

All cases and controls were female and had four Han Chinese grandparents. Cases and controls were excluded if they had a pre-existing history of bipolar disorder, any type of psychosis or mental retardation. Cases were between 30 and 60 years old, had two or more episodes of MD, with the first episode occurring between ages 14 and 50, and had not abused drugs or alcohol prior to their first depressive episode. Controls were chosen to match the region of origin of cases, were aged between 40 and 60, had never experienced an episode of MD and were not blood relatives of cases. An older minimal age of controls was used to reduce the chances that they might have a subsequent first onset of MD. The mean (S.D.) age of cases and controls in the dataset was similar: 44.4 (8.9) years for cases and 47.7 (5.6) years for controls.

All subjects were interviewed using a computerized assessment system. All interviewers were medical professionals and were trained by the CONVERGE team for a minimum of 1 week in the use of the interview. The interview includes assessment of demographic factors, psychopathology, psychosocial functioning and personal characteristics. Interviews were tape recorded and a proportion of them were listened to by the trained editors who provided feedback on the quality of the interviews. The study protocol was approved centrally by the Ethical Review Board of Oxford University and the ethics committee in participating hospitals in China.

### Measures

The diagnoses of lifetime MD was established with the Composite International Diagnostic Interview (CIDI; [22]), which classifies diagnoses according to DSM-IV criteria [23]. The interview was originally translated into Mandarin by a team of psychiatrists in Shanghai Mental Health Centre, with the translation reviewed and modified by members of the CONVERGE team.

Additional information using instruments developed for the Virginia Adult Twin Study of Psychiatric and Substance Use Disorders (VATSPSUD; [24]), translated and reviewed for accuracy by members of the CONVERGE team, was collected on stressful life events, CSA, family history and parent–child relationships etc. The stressful life events section assessed 16 traumatic lifetime events and the age of their occurrence. The CSA module was a shortened version of the more detailed module used in the VATSPSUD study, which is in turn based on the instrument developed by Martin et al. [25]. We assessed separately the history of MD in mothers and fathers of our cases and controls using the Family History Research Diagnostic criteria [26], and parent–child relationships were measured with the 16-item Parental Bonding Instrument (PBI) modified by Kendler [27] based on Parker’s original 25-item instrument [28]. Three factors were extracted from these 16 items and labeled warmth, protectiveness and authoritarianism.

The computerized interview system is described elsewhere [21].

Symptoms reported during the most severe MD episode were classified according to DSM-IV diagnostic “A criteria” such as loss of interest, weight loss/gain, insomnia/hypersomnia, etc.

There is evidence that sensitive subjects such as CSA are more accurately reported with more confidential methods of assessment [29], and therefore participants were asked to fill in a paper questionnaire about CSA [25]. The questions asked whether, before the subject was 16 years old, did any adult or any other older person involve the subject in any unwanted incidents such as (1) inviting or requesting them to do something sexual, (2) kissing or hugging in a sexual way, (3) touching or fondling private parts, (4) showing their sex organs, (5) making them touch the person in a sexual way, or (6) attempting or having sexual intercourse. The possible responses were ‘never’, ‘once’ and ‘more than once’. We used these responses to define three forms of CSA [5]: (1) non-genital CSA including sexual invitation, sexual kissing, and exposing (2) genital CSA including fondling and sexual touching and (3) attempted or completed intercourse.

We assessed childhood physical abuse (CPA) through the question in our stressful life events section: ‘Were you ever physically abused as a child?’ We assessed childhood emotional neglect (CEN) through the question in this section: ‘Were you ever seriously neglected as a child?’ Emotional neglect refers to a lack of emotional support and inadequate attention to a child’s emotional needs, including the need for affection. Physical abuse refers to bodily assaults on a child by an older person that pose a risk of, or result in, injury.

Thirty-nine participants (6.4%) in the rMD group and 26 participants (17.2%) in the control group were more than 16 years old when sexual abuse first occurred (Chi square test, p<0.001). These subjects were not incluced as CSA cases.

### Statistical analysis

We examined the association between CSA and rMD using logistic regression in R [30], from which we derived estimates of odds ratios (ORs) and their associated 95% confidence intervals (CIs). We examined the degree to which the association between self-reported CSA and rMD changed with the inclusion of variables that reflect parental family history of depression, CPA, CEN and parent–child relationships according to the PBI. We controlled only for age, social class, occupation and education in the first step of the logistic regression model, and then for family history of MD in the second step. In the third step, we added CPA, CEN and PBI. The above analysis was carried out in samples recruited before June 2010 (Cohort 1, MD n = 1970, controls n = 2597) and after June 2010 (Cohort 2, MD n = 4169, controls n = 3473). Logistic regression was carried out in three steps to analyze the effect of cohort and the interaction between cohort and CSA. To examine the relationship between CSA and MD co-morbidity, we predicted, in case only, the risk of depressive patients with CSA having dysthymia, generalized anxiety disorder (GAD), panic disorder (PD), postnatal depression and phobia with MD, building logistic regression models in three steps.

The impact of the nine DSM-IV criteria depressive symptoms on CSA were assessed by logistic regression. The association between specific features of CSA and rMD was also assessed by logistic regression. Associations between variables were expressed as ORs and 95% CIs. The age at which CSA occurred in rMD and non-MD participants was analyzed by Student’s *t*-test. R [30] was used in data analysis.

## Results

### Prevalence of CSA

Data on CSA were obtained from 5,295 cases of recurrent MD and 5,820 controls. Table 1 shows the prevalence of specific forms of CSA in cases versus controls in our preliminary and final samples. The rates of the specific forms of CSA were consistently higher in cases versus controls in two cohorts. In the combined sample, any form of CSA was reported by 10.6% of the women with a history of rMD versus 2.1% of controls. Unwanted attempted or completed intercourse before the age of 16 was reported by 3.0% of women with rMD versus 0.3% of controls in full samples.

**Table 1:** Childhood sexual abuse (CSA) of all participants.

	Any CSA	Non-genital	Genital	Intercourse
	Cohort 1			
MD (n = 1920)	189 (9.8%)***	67 (3.5%)***	76 (4.0%)***	46 (2.4%)***
Controls (n = 2588)	70(2.7%)	31(1.2%)	29 (1.1%)	10 (0.4%)
	Cohort 2			
MD(n = 4041)	392(9.7)***	108(2.7)***	171(4.2)***	113(2.8)***
Controls(n = 3439)	68(2.0)	42(1.2)	19(0.6)	7(0.2)
	Combined			
MD(n = 5295)	560(10.6)***	167(3.2)***	237(4.5)***	156(3.0)***
Controls(n = 5820)	125(2.1)	66(1.1)	44(0.7)	15(0.3)

Values are given as n (%).

*** *p*<0.001.

We analyzed the association between CSA and rMD in cohort 1 and cohort 2 and found no significant differences (*p* > 0.05) between results from the two cohorts (Table 2). Indeed, the results showed remarkable consistency. The consistency justified combining the entire sample. In the rest of this paper, all results are given for the complete sample.

**Table 2:** Childhood sexual abuse (CSA) and the odds ratios for major depression (MD).

	Cohort 1	Cohort 2	P value of difference	Combined
Any CSA Model 1	3.26(1.95–5.45)***	4.06(3.10–5.40)***	0.696	4.06(3.19–5.24)***
Model 2	2.63(1.55–4.46)***	3.79(2.80–4.95)***	0.555	3.62(2.82–4.70)***
Model 3	1.86(1.06–3.29)*	2.06(1.45–2.95)***	0.843	2.17(1.60–2.98)***
Non-genital Model 1	2.47(1.17–5.23)*	2.01(1.38–2.99)***	0.522	2.21(1.58–3.15)***
Model 2	1.91(0.88–4.16)	1.96(1.32–2.95)***	0.857	2.01(1.42–2.90)***
Model 3	1.47(0.66–3.29)	1.38(0.84–2.30)	0.980	1.44(0.93–2.26)
Genital Model 1	2.77(1.32–5.83)**	5.82(3.69–9.74)***	0.212	5.24(3.52–8.15)***
Model 2	2.31(1.07–4.98)*	5.32(3.33–8.98)***	0.222	4.77(3.17–7.48)***
Model 3	1.57(0.66–3.74)	3.22(1.80–6.15)***	0.387	3.09(1.88–5.35)***
Intercourse Model 1	13.35(1.83–97.42)*	10.39(5.18–24.73)***	0.906	10.65(5.56–23.71)***
Model 2	10.87(1.47–80.38)*	8.73(4.30–20.94)***	0.899	9.02(4.67–20.22)***
Model 3	6.99(0.92–53.22)	2.45(1.10–6.23)*	0.552	3.07(1.49–7.18)**

Values are given as odds ratio (95% confidence interval).

Model 1 includes control variables for age and educational background. Model 2 includes in addition parental family history of depression. Model 3 includes in parent–child relationship as assessed by the the PIB, childhood physical abuse (CPA) and childhood emotional neglect (CEN).

* *p*<0.05, ** *p*<0.01, *** *p*<0.001.

Cohort 1: MD n = 1920, Controls n = 2588; Cohort 2: MD n = 4041, Controls n = 3439; Combined: MD n = 5295, Controls n = 5820.

While all attempts were made to match cases and controls, the groups differed in a number of features, previously reported as risk factors for MD. In general, cases had less education, and lower social and occupational status than controls. These, and related features are described in detail in other publications [31], [32], [33], [34]. For the analyses, reported in this paper, we controlled for social, occupational and educational differences where necessary.

### The relationship between rMD and CSA

Any form of CSA was strongly associated with a history of rMD (OR 4.06)(model 1 in Table 2). Looking at specific forms of CSA, the association with rMD strengthened with increasingly severe abuse: non-genital (OR 2.21), genital (OR 5.24) and intercourse (OR 10.65). We considered whether the relationship between CSA and rMD reflected the presence of mental illness in the parents. We therefore repeated the analyses, adding in family history diagnoses of mother and father (model 2 in Table 2). The magnitude of the association between CSA and rMD decreased, but remained significant.

CSA is also correlated with childhood adversity, in particular CPA and CEN, and with the nature of the relationship between parent and child. To explore whether parent–child relationships and childhood adversity explain the association between CSA and rMD, we added into model 3 (from Table 2) the results from the PBI, and the assessments of CPA and CEN. We found that the OR for the effect of CSA on rMD was decreased (Table 2). That is, controlling for the parent–child relationship, CPA and CEN, any form of CSA, genital CSA, and intercourse CSA remained significantly associated with a history of rMD in our sample.

### The relationship between the CSA and comorbidity of rMD and phenomenology of rMD

We next considered the possibility that the experience of CSA could increase the rate of comorbid disorders in patients with rMD. We examined the association between self-reported CSA and major depression with dysthymia, postnatal depression, generalized anxiety disorder (GAD), panic disorder (PD) and phobia. Controlling only for age and education (table 3, model 1) a lifetime history of GAD, dysthymia, panic disorder, postnatal depression and phobia was associated with CSA (*p*<0.05). These associations all remained significant with the inclusion of parental MD history (table 3, model 2). Adding PBI, CPA and CEN into the model, the association between CSA and dysthymia and between CSA and phobia remained significant (*p*<0.05).

**Table 3:** Childhood sexual abuse (CSA) and the odds ratios for co-morbid disorders.

	Any CSA	No CSA	Model 1	Model 2	Model 3
MD and dysthymia (n = 588)	107(18.2%)	465 (79.1%)	2.22 (1.73–2.83) ***	2.13 (1.66–2.72) ***	1.60(1.11–2.27)*
MD and postnatal depression (n = 982)	144(14.7%)	829(84.4%)	1.43 (1.14–1.79) **	1.39 (1.11–1.74) **	1.35(0.99–1.83)
MD and GAD (n = 1516)	178 (11.7%)	1309 (86.3%)	1.28 (1.05–1.55) *	1.24(1.02–1.51) *	1.06 (0.81–1.39)
MD and PD (n = 400)	56(14.0%)	336 (84.0%)	1.74 (1.26–2.36) ***	1.68 (1.22–2.28) **	1.39(0.88–2.12)
MD and phobia (n = 2333)	273(11.7%)	2036(87.3%)	1.46 (1.22–1.75) ***	1.40 (1.16–1.68) ***	1.41(1.09–1.80)**

MD, major depression; GAD, generalized anxiety disorder; PD, panic disorder.

Values are given as n (%) or odds ratio (95% confidence interval).

Model 1 includes control variables for age and educational background. Model 2 includes in addition parental family history of depression. Model 3 includes in parent–child relationship as assessed by the PIB, childhood physical abuse (CPA) and childhood emotional neglect (CEN).

* *p*<0.05, ** *p*<0.01, *** *p*<0.001.

Within cases (table 4, model 1), we found that depressed patients with CSA had a significantly earlier age of onset of MD. This result was significant when we included parental history of MD (model 2) and when including parental child relation, CPA and CPN in model 3. The number of reported depressive episodes for those with CSA was significantly more than those without CSA in model 1 and model 2. In model 3 the relationship was not significant. The duration of the longest episode of MD for those with CSA was significantly greater than those without CSA and this was seen in all models.

**Table 4:** The relationship between childhood sexual abuse (CSA) and clinical features of major depression (MD).

	Age of onset of MD^a^			Numbers of episodes of MD^b^			Duration of the longest episode of MD^c^		
	Model1	Model 2	Model 3	Model1	Model 2	Model 3	Model1	Model 2	Model 3
Coefficients	–1.89	–1.80	–1.27	0.095	0.086	–0.031	0.307	0.301	0.231
*p*-value	9.61e-08	3.63e-07	0.005	5.80e-06	4.01e-05	0.291	<2e-16	<2e-16	<2e-16

Model 1 includes variables that reflect age and educational background. Model 2 includes in addition parental family history of depression. Model 3 includes in addition parent–child relationship, childhood physical abuse (CPA) and childhood emotional neglect CEN).

a The results from a linear regression model with age of onset of MD as response and with ‘any CSA’ as predictor.

b The results from a Poisson regression model with numbers of episodes of MD as response and with ‘any CSA’ as predictor.

c The results from a Poisson regression model with duration of the longest episodes of MD as response and ‘any CSA’ as predictor.

Table 5 summarizes the association between CSA and individual DSM-IV criteria for MD reported at the worst lifetime episode. Any form of CSA was significantly associated with suicidal ideation or attempt (OR 1.50) and feelings of worthlessness or guilt (OR 1.41). Looking at specific forms of CSA, the association with genital (OR 1.66) and intercourse (OR 1.69) was only significant with suicidal ideation or attempt.

**Table 5:** Association of Childhood sexual abuse (CSA) major depression (MD) symptoms.

	Any CSA	Non-genital	Genital	Intercourse
Depressed mood	1.95(0.56–12.33)	0.57(0.16–3.65)	2.05E+6(5.12E-11-NA)	2.14E+6(7.22E-15-NA)
Loss of interest	1.78(0.71–5.97)	0.72(0.26–3.01)	2.97(0.63–52.92)	1.50E+6(0.0007-4.36E+83)
Weight loss/gain, appetite changes	0.93(0.70–1.25)	0.85(0.54–1.40)	1.19(0.77–1.94)	0.73(0.46–1.21)
Insomnia/hypersomnia	1.05(0.71–1.60)	1.37(0.68–3.29)	0.73(0.45–1.25)	1.69(0.76–4.83)
Psychomotor agitation/retardation	1.15(0.86–1.58)	1.17(0.71–2.03)	1.02(0.68–1.60)	1.44(0.82–2.77)
Fatigue/loss of energy	0.93(0.67–1.32)	1.47(0.78–3.16)	0.82(0.52–1.34)	0.77(0.45–1.43)
Feelings of worthlessness/guilt	1.41(1.02–2.02)*	1.09(0.66–1.92)	1.68(1.01–3.00)	1.58(0.87–3.24)
Concentrate/indecisiveness	1.31(0.72–2.63)	0.62(0.30–1.51)	2.04(0.75–8.42)	3.90(0.86–69.00)
Suicidal ideation/attempt	1.50(1.20–1.89)***	1.21(0.85–1.77)	1.66(1.19–2.35)**	1.69(1.12–2.63)*

Values are given as odds ratio (95% confidence interval).

* *p*<0.05, ** *p*<0.01, *** *p*<0.001.

### Features of CSA that increase risk of rMD

The mean age of first reported CSA in rMD patients 10.99 (sd 3.55), and 11.75 (sd 3.49) for the controls. The difference between two groups was significant (*t* = –2.19, *df* = 185.802, *p* = 0.031). All other CSA characteristics including attempted or completed intercourse, age of perpetrator, gender of perpetrator, abuse by a relative, whether forced or threatened, how affected at the time, of individuals with rMD versus controls were assessed by logistic regression (results showed in table 6). Only the attempted or completed intercourse (OR 3.47, 95%CI 1.66–8.22, *p*<0.01), use of force and threats (OR 1.95, 95%CI 1.05–3.82, *p*<0.05) and how strongly the victims were affected at the time (OR 1.39, 95%CI 1.20–1.64, *p*<0.001) were associated with risk for rMD.

**Table 6:** Characteristics of sexual abuse in major depression (MD) patients and controls who reported childhood sexual abuse (CSA).

	MD N = 560	Controls N = 125	OR (95%CI)/*t*
Age at time of abuse	10.99(3.55)	11.75(3.49)	*t* = –2.19, *df* = 185.802, *p* = 0.031
Intercourse vs others	156/404	15/110	3.47(1.66–8.22)**
Age of perpetrator			0.96(0.77–1.19)
Under 15 years	13	70	
15–18 years	22	104	
19–24 years	27	110	
25–49 years	53	234	
Older than 50 years	10	42	
Male/female or both	39/521	9/116	1.30(0.47–4.62)
Relative or not	187/373	32/93	1.57(0.87–2.98)
Forced or not	187/373	24/101	1.95(1.05–3.82)*
How affected			1.39(1.20–1.64)***
1	188	65	
2	83	23	
3	68	16	
4	43	7	
5	50	5	
6	33	4	
7	95	5	

Values are given as n (%) and odds ratio (95% confidence interval).

* *p*<0.05, ** *p*<0.01, *** *p*<0.001.

## Discussion

Our study produced four major findings. First, we were able to replicate the findings from analysis of the first 2,000 cases of the CONVERGE study. We found in Han Chinese women that CSA is robustly associated with an increased risk of developing rMD. This increases our confidence in the validity of this important finding. Second, CSA affected the clinical features of rMD. In women with rMD, those with a history of CSA had an earlier age of onset, longer depressive episodes and an increased risk to suffer from dysthymia and phobia. Third, we found that any form of CSA was associated with suicidal ideation or attempt and feelings of worthlessness or guilt. Genital and intercourse forms of CSA were associated with suicidal ideation or attempt. Fourth, the use of force or threats, the magnitude of the upset experienced by the subject at the time of abuse, and younger age at CSA were significantly associated with rMD.

In our full sample, any form of CSA was reported by 10.6% of the women with a history of rMD versus 2.1% of controls. This frequency of CSA is lower than in other Chinese studies, in which at least one type of CSA has been reported in 16.7% to 22.1% of female students [35], [36], [37]. However, our study differs in that we studied an older group of women (the mean age of our sample is 46 years). The marked economic and cultural changes occurring in China may be one of the reasons for the different rates.

The association of CSA with the risk of MD has been extensively documented in Western populations [5], [12], [38], [39], [40], [41], [42], as has the dose–response relationship between CSA and risk for developing MD [38], [39], and the finding that CSA involving intercourse has the largest odds ratio [12], . Our findings indicate that these observations about the relationship between MD and CSA also hold true in China. Our findings are likely to be robust because of the large sample we have surveyed: the largest sample previously studied included 3982 subjects [38].

On average, parents with psychiatric illness provide poorer environments for their children, potentially not attending to their daughters’ wellbeing and so increasing the risk of abuse. This means CSA could be an indirect or a direct manifestation of parental depressive disorder. In our sample, any form of CSA was significantly associated with a parental history of depression (OR 3.51, 95% CI 2.90–4.23, *p*<0.001). When we controlled for parental MD history, the association between CSA and rMD decreased mildly.

Some researchers have reported that poor parental care, high control, overprotection, and alienation in the relationship between parent and child increases the risk of developing MD [43], [44], [45], [46], [47], [48], [49], [50], [51], [52]. In our sample, any form of CSA was significantly associated with perceived parenting. Others studies obtained significant associations between CPA, CEA and MD [43], [53], [54], [55], [56]. Some reported that CPA and CEN in part explain the effect of CSA on MD [12], [39], [40]. There was some evidence that CEN is more strongly related to the presence of MD in adulthood than either CPA or CSA [57]. One study showed that childhood adversities were highly interrelated [58]. In our sample, CEN (OR 6.58, 95% CI 5.39–8.00, *p*<0.001) and CPA (OR 7.88, 95% CI 6.04–10.19, *p*<0.001) were strongly related. When we controlled for the effect of parent–child relationship, CPA and CEN, we found that the strength of the relationship between the risk of rMD and any CSA remained significant, but the OR decreased substantially, especially for intercourse (from 10.39 to 2.45) in cohort 2. This suggested that part of the impact of CSA on risk for rMD is a relatively non-specific index of disruption and poor treatment in the home of origin. However a substantial part of the effect appears to be specific to CSA itself.

Our results showed that increasing severity of abuse is associated with an increased risk of rMD. CSA had a systematic ‘dose–response’ relationship with risk for rMD: the greater the severity of CSA, the stronger is the observed association with rMD. Controlling for potential confounders (specifically parental history of MD, parent–child relationship and other childhood adversities) attenuated the CSA–MD association but the association between rMD and ‘any CSA’, genital, and intercourse still remained significant. This suggests that CSA in part causes rMD. When controlled for the family history of depression, parent-child relationship, CPA, and CEN, the ORs decreased. It should be noted that exposure to multiple forms of abuse, including physical and emotional neglect, also increases the risk of rMD. Thus, although the evidence seems to be consistent with a causal model, we cannot exclude more complex relationships between CSA and rMD.

We also considered whether the effect of CSA on rMD might result in characteristic symptomatology. The greater prevalence of co-morbid dysthymia and phobia, together with an earlier age of onset and longer episodes, indicates that this may be true. Some studies found that MD patients, with a history of CSA, had more severe depressive symptoms, longer duration of index depressive episode, earlier onset, and higher comorbidity [16], [17], [18], [19]. Our findings were consistent with these studies.

Significant CSA-associated risk for suicidal thoughts and behavior has been reported in non-clinical, twins, population-representative adolescent cohorts, students, patients in primary care, and other clinical populations [38], [41], [42], [43], [44], [45], [46], [47], [48], [49], [50], [51]. Fewer studies have examined CSA as a risk factor for suicidal thoughts and attempt in MD patients. Sarchiapone et al. (2007) reported that childhood trauma was significantly associated with making a suicide attempt in unipolar depression patients [52]. Our research found that CSA, especially “genital” and “intercourse”, increased the risk of having suicidal ideation or attempt in female Chinese rMD patients.

Some studies demonstrated a positive association between shame, poor or impaired self-esteem and childhood abuse [53], [54], [55], [56]. In our study, we found a significant relationship between CSA and feelings of worthlessness or guilt. Our result is consistent with these studies.

Many aspect of CSA may have a role in the pathogenesis of MD. Few studies have so far examined the relationship between characteristics of CSA and MD. One study identified a number of CSA features that increased the risk of MD: these were the age of abuse occurrence, the presence of attempted or completed intercourse, more than one perpetrator, gender of perpetrator, abuse by a relative, forced or threatened abuse, how strongly the victims were affected at that time [59]. Another study found that greater numbers of CEA and CSA perpetrators were associated with a greater number of depressive episodes in adulthood [60]. Age of onset has been significantly linked to depression by some investigators; an early age of onset predicted depression [61], [62], [63]. Abuse perpetrated by a father figure has also been significantly related to the presence of depression in adult survivors [57]. Mennen and Meadow (1995) found that force used by a non-father figure was significantly related to increased rates of depression in sexually abused girls [64]. In our study, we also found that earlier age onset of CSA was associated with rMD. Greater effect at that time and the use of force by perpetrator predicted rMD. These findings are consistent with previous findings.

Our study has several potential limitations. First, as in most epidemiological studies, CSA in our sample was assessed retrospectively. Recall may be inaccurate and/or biased [49], [65]. Estimates for the rates of CSA in our control sample (2.64% for any CSA and only 0.30% for unwanted intercourse) are lower than estimates obtained from other, non-Chinese samples (24.7–30.4% for any CSA and 5.6–8.4% for intercourse) [5], [38], [66]. These low rates may reflect either under-reporting in Chinese populations or truly lower rates of CSA in China versus most Western populations.

Subjects reported CSA in a self-report questionnaire, so the low rates of CSA in our sample were not the result of hesitance to admit to CSA during a face-to-face interview. If cultural factors influenced subjects to underreport CSA, the general expectation is that this bias would impact equally on our cases and controls. However, we cannot rule out the possibility that our cases (because of depressed mood or greater contact with health professionals) were more willing to report CSA than were our controls. In our sample, more participants in the control group (17.2%) than in the rMD group (6.4%) were older than 16 at first abuse. Although, analysis excluding or these participants yielded same results, we cannot exclude the possibility that our observed association between CSA and rMD could arise from biased reporting rather than a true causal association.

Second, our assessments of CEN and CPA were obtained from a single item. Despite covering important potential traumas, our coverage was far from exhaustive. It is possible that our aspects of the home environment predisposed to both CSA and rMD, thereby biasing upwards our estimates of their association.

Third, our assessment of socio-economic status was based on the highest educational attainment of study participants. Various markers of familial dysfunction are associated with MD in adult. We did not assess additional family characteristics, such as personality and cognitive abilities, which might confound the relationship between CSA and MD.

Fourth, our findings may not apply to other groups. Our cases were obtained from subjects attending psychiatric clinics in general hospitals, and this population may differ from the population undergoing minor surgery in general hospitals or attending community centers. In our study, 63% of of control were recruited from community centers. We observed no differences in demographic features between controls obtained in community centres and in hospitals but it is possible that some unacknowledged differences within the control groups contribute to our findings.

Fifth, our sample had recurrent MD. The impact of CSA on individuals with a single episode of MD who do not present for treatment may be different; we do not know whether our results generalize to other groups of MD patients.

In summary, we have found that CSA is strongly associated with rMD in Chinese women. The association shows a strong dose–response relationship and is mildly attenuated when controls are added for parental depression and other childhood environmental adversities. These results suggest, but do not prove, that the CSA–MD association in China is causal, as has been suggested in US and Australian samples [5], [38]. We also found that CSA was associated with suicide ideation or attempt and some subtype of MD in female patients; early age of onset, being forced and greater affected at the time of CSA was more likely to have rMD.

It is important for healthcare practitioners to know that CSA could increase the risk of developing MD. When dealing with a patient with CSA, clinicians should assess the relevance of such a history and address it therapeutically, as this may be helpful for the patient.

### Ethics statement

The study protocol was approved centrally by the Ethical Review Board of Oxford niversity (Oxford Tropical Research Ethics Committee) and the ethics committees responsible for the follow hospitals in China: No. 4 Affiliated Hospital of Jiangsu University, No.20 Zhengdong Road, Zhenjiang, Jiangsu, P.R.C.; Shanghai Mental Health Center, No.600 South Wanping Road, Shanghai, P.R.C.; Huashan Hospital of Fudan University, No.12 Middle Wulumuqi Road, Shanghai, P.R.C.; Chinese Traditional Hospital of Zhejiang, No.54 Youdian Road, Hangzhou, Zhejiang, P.R.C.; No.1 Hospital of Zhengzhou University, No.1 East Jianshe Road, Zhengzhou, Henan, P.R.C.; Xinhua Hospital of Zhejiang Province, No.318 Chaowang Road, Hangzhou, Zhejiang, P.R.C.; No.1 Hospital of Shanxi Medical University, No.85 South Jiefang Road, Taiyuan, Shanxi, P.R.C.; ShengJing Hospital of China Medical University, No.36 Sanhao Street, Heping District, Shenyang, Liaoning, P.R.C.; No. 1 Hospital of Medical College of Xian Jiaotong University, No.277 West Yanta Road, Xian, Shaanxi, P.R.C.; Jilin Brain Hospital, No.98 West Zhongyang Road, Siping, Jilin, P.R.C.; Mental Hospital of Jiangxi Province, No.43 Shangfang Road, Nanchang, Jiangxi, P.R.C.; Xian Mental Health Center, No.15 Yanyin Road, New Qujiang District, Xian, Shaanxi, P.R.C.; Beijing Anding Hospital of Capital University of Medical Sciences, No.5 Ankang Hutong, Deshengmen wai, Xicheng District, Beijing, P.R.C.; Shandong Mental Health Center, No.49 East Wenhua Road, Jinan, Shandong, P.R.C.; No. 3 Hospital of Sun Yat-sen University, No.600 Tianhe Road, Tianhe District, Guangzhou, Guangdong, P.R.C.; Hebei Mental Health Center, No.572 Dongfeng Road, Baoding, Hebei, P.R.C.; Chongqing Mental Health Center, No.102 Jinzishan,Jiangbei District, Chongqing, P.R.C.; Tianjin Anding Hospital, No.13 Liulin Road, Hexi District, Tianjin, P.R.C.; The First Hospital of China Medical University, No.155 North Nanjing Street, Heping District, Shenyang, Liaoning, P.R.C.; Psychiatric Hospital of Henan Province, No.388 Middle Jianshe Road, Xinxiang, Henan, P.R.C.; Nanjing Brain Hospital, No.264 Guangzhou Road, Nanjing, Jiangsu, P.R.C.; Harbin Medical University, No.23 Youzheng street, Nangang District, Haerbin, Heilongjiang, P.R.C.; Shenzhen Kang Ning Hospital, No.1080, Cuizhu Street, Luohu District, Shenzhen, Guangdong, P.R.C.; First Hospital of Hebei Medical University, No.89 Donggang Road,, Shijiazhuang, Hebei, P.R.C.; Guangzhou Brain Hospital (Guangzhou Psychiatric Hospital), No.36 Mingxin Road, Fangcun Avenue, Liwan District, Guangzhou, Guangdong, P.R.C.; No.1 Hospital of Chongqing Medical University, No.1 Youyi Road,Yuanjiagang,Yuzhong District, Chongqing, P.R.C.; Dalian No.7 Hospital, No.179 Lingshui Road, Ganjingzi District, Dalian, Liaoning, P.R.C.; No.3 Hospital of Heilongjiang Province, No.135 Jiaotong Road, Beian, Heilongjiang, P.R.C.; Wuhan Mental Health Center, No.70, Youyi Road, Wuhan, Hubei, P.R.C.; Sichuan Mental Health Center, No.190, East Jiannan Road, Mianyang, Sichuan, P.R.C.; Mental Health Institute of Jining Medical College, Dai Zhuang, Bei Jiao, Jining, Shandong, P.R.C.; Liaocheng No.4 Hospital, No.47 North Huayuan Road, Liaocheng, Shandong, P.R.C.; Mental Health Center of West China Hospital of Sichuan University, No.28 South Dianxin Street, Wuhou District, Chengdu, Sichuan, P.R.C.; Suzhou Guangji Hospital, No.286, Guangji Road, Suzhou, Jiangsu, P.R.C.; Anhui Mental Health Center, No.316 Huangshan Road, Hefei, Anhui, P.R.C.; Ningbo Kang Ning Hospital, No.1 Zhuangyu Road,Zhenhai District, Ningbo, Zhejiang, P.R.C.; Changchun Mental Hospital, No.4596 Beihuan Road, Changchun, Jilin, P.R.C.; No.2 Hospital of Lanzhou University, No.82, Cuiyingmen, Lanzhou, Gansu, P.R.C.; Fuzhou Psychiatric Hospital, No.451 South Erhuan Road,Cangshan District, Fuzhou, Fujian, P.R.C.; Harbin No.1 Special Hospital, No.217 Hongwei Road, Haerbin, Heilongjiang, P.R.C.; Jining Psychiatric Hospital, North Dai Zhuang,Rencheng District, Jining, Shandong, P.R.C.; No.2 Xiangya Hospital of Zhongnan University, No.139 Middle Renmin Road,Furong District, Changsha, Hunan, P.R.C.; Xijing Hospital of No.4 Military Medical University, No.17 West Changle Road, Xian, Shaanxi, P.R.C.; Mental Health Center of Shantou University, No.243 Daxue Road, Shantou, Guangdong, P.R.C.; Tongji University Hospital, No.389 Xinchun Road, Shanghai, P.R.C.; Huaian No.3 Hospital, No.272 West Huaihai Road, Huaian, Jiangsu, P.R.C.; Huzhou No.3 Hospital, No.255 Gongyuan Road, Huzhou, Zhejiang, P.R.C.; Mudanjiang Psychiatric Hospital of Heilongjiang Province, Xinglong, Mudanjiang, Heilongjiang, P.R.C.; No.1 Hospital of Jinan University, No.613 West Huangpu Avenue, Guangzhou, Guangdong, P.R.C.; Qingdao Mental Health Center, No.299 Nanjing Road, Shibei District, Qingdao, Shandong, P.R.C.; Guangxi Longquanshan Hospital, No.1 Jila Road, Yufeng District, Liuzhou, P.R.C.; Daqing No.3 Hospital of Heilongjiang Province, No.54 Xitai Road, Ranghulu district, Daqing, Heilongjiang, P.R.C.; Tangshan No.5 Hospital, No.57 West Nanxin Road, Lunan District, Tangshan, Hebei, P.R.C.; Anshan Psychiatric Rehabilitation Hospital, No.127 Shuangshan Road,Lishan District, Anshan, Liaoning, P.R.C.; Weihai Mental Health Center, Qilu Avenue, ETDZ, Weihai, Shandong, P.R.C.; Renmin Hospital of Wuhan University, No.238 Jiefang Road, Wuchang District, Wuhan, Hubei, P.R.C.; Tianjin First Center Hospital, No.55 Xuetang Street, Xinkai Road, Hedong District, Tianjin, P.R.C.; Hainan Anning Hospital, No.10 East Nanhai Avenue, Haikou, Hainan, P.R.C.

Major psychotic illness was an exclusion criterion. All interviewers were mental health professionals who are well able to judge decisional capacity. All participants provided written informed consent before interview. The study posed minimal risk (an interview and saliva sample).
